# Kuroshio Corridor: larval dispersal networks explain geographically independent connectivity among coral habitats in Japan

**DOI:** 10.1038/s41598-026-40448-z

**Published:** 2026-02-20

**Authors:** Naoki Saito, Hiroki Kise, Yuichi Nakajima, Akira Iguchi

**Affiliations:** 1 Integrated Research Center for Nature Positive Technology, National Institute of Advanced Industrial Science and Technology (AIST), Tsukuba, Japan; 2 Geological Survey of Japan, National Institute of Advanced Industrial Science and Technology (AIST), Tsukuba, Japan; 3 Fisheries Technology Institute, Japan Fisheries Research and Education Agency, Kamisu, Japan

**Keywords:** Coral reef, Ecological network, Gene flow, Genetic connectivity, Graph theory, Kuroshio Current, Ecology, Ecological networks, Molecular ecology, Ocean sciences, Marine biology, Physical oceanography

## Abstract

Effective conservation of patchy marine habitats, such as those around islands, requires an understanding of ecological networks maintained through larval dispersal. In the Nansei Islands of Japan, previous studies suggested that the genetic connectivity of reef-building corals was independent of geographic distance along this near-linear island chain, likely due to the Kuroshio Current. However, this has not yet been confirmed using an integrated approach combining population genetics and physical oceanography. Here, we conducted biophysical modelling of larval dispersal and population genomic analysis across the Nansei Islands for a major reef-building coral, *Acropora digitifera*. Modelled larval dispersal networks showed that dispersal from the southern to northern ends of the archipelago occurred more than six times as frequently as dispersal to the central islands. Genetic differentiation between sites showed a stronger association with modelled dispersal (*R* = -0.44) than with geographic distance (*R* = 0.26). Kume Island served as a key stepping stone in the larval dispersal network, despite being outside the area’s national parks. This study provides fundamental insights into the dispersal pathway connecting distant locations along the Kuroshio Current—which we refer to as the ‘Kuroshio Corridor’—a concept that is crucial for connectivity-based conservation.

**Supplementary Information:**

The online version contains supplementary material available at 10.1038/s41598-026-40448-z.

## Introduction

Ecological networks of patchy habitats, such as islands, linked by individual movement pathways provide a foundation for effective conservation^1–3^. Maintaining ecological networks facilitates the influx of individuals from other habitats, supporting population recovery following disturbances^4,5^ and preserving genetic diversity^6,7^. For many marine organisms, movement among distant habitats occurs through dispersal during the pelagic larval stage^8,9^. Therefore, identifying larval dispersal pathways and sink-source relationships among habitats is key for successful marine conservation^3,4^. For example, marine protected areas in the Great Barrier Reef^10,11^ and the North American Channel Islands^12^ have been shown to form ecological networks, as evidenced by biophysical larval dispersal models and population genetic analyses.

**Fig. 1:**
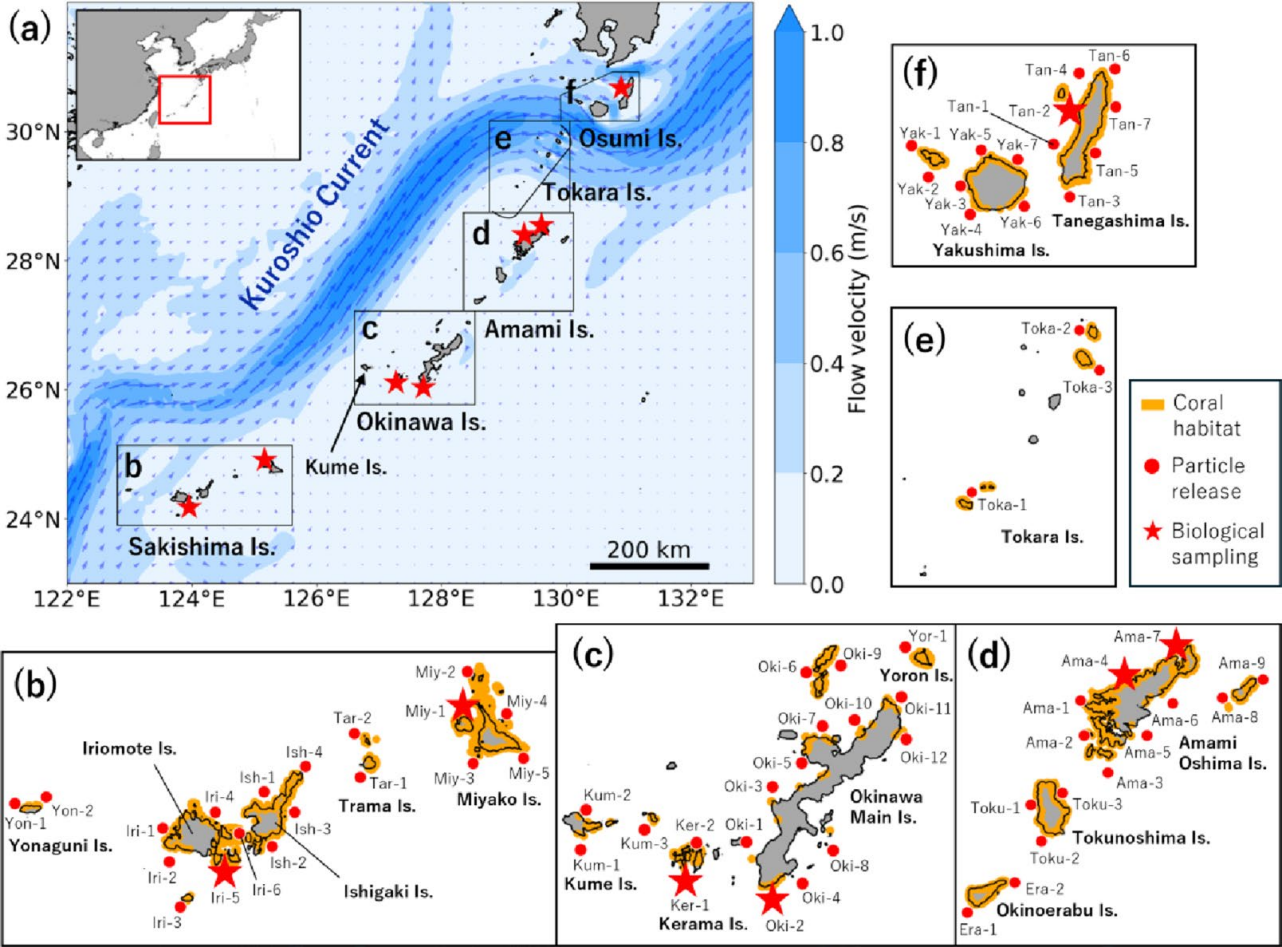
Study area. Red dots represent particle release sites in the biophysical model, and stars mark biological sampling sites. All biological sampling sites also served as particle release sites. In (**a**), blue shading represents the 5-year (2019–2023) mean current velocities at 0 m depth during the coral spawning period (May to August), derived from the ocean model JCOPE-T, and arrows indicate the current directions. In (**b**–**d**), orange areas represent coral habitats surveyed by the Japanese Ministry of the Environment. The coastline was visualised using the GEBCO_2022 Grid^65^ for topographic data with the Python package ‘rioxarray’ (https://github.com/corteva/rioxarray, accessed 6th February 2026).

The Nansei Islands in southern Japan (Fig. 1) host some of the world’s most biodiverse coral reefs^13,14^, and many of the islands have been designated as national parks. However, the region also contains some of the most threatened coral reefs globally^13,14^. Population genetics studies on broadcast-spawning reef-building corals (hereafter ‘corals’) have reported high genetic connectivity throughout the archipelago, which spans approximately 1000 km^15–18^. Given that the typical larval dispersal distance of corals is estimated to be 50–150 km^8^, the extent of genetic connectivity in the Nansei Islands is remarkably broad. Furthermore, despite the archipelago’s nearly linear geographic configuration, relatively high genetic connectivity has been observed between its southernmost and northernmost islands^15,18^. This unexpectedly high genetic connectivity has been interpreted to reflect the effects of the nearby Kuroshio Current on larval dispersal^15–18^, although these findings have been based solely on comparisons among a limited number of sampling sites. Furthermore, few studies have quantitatively compared genetic connectivity among coral habitats with larval dispersal estimates derived from biophysical modelling in the region^19^.

Ocean currents, among other factors, shape larval dispersal pathways, sometimes resulting in genetic connectivity that does not follow geographic distance. In some archipelagos, where islands are separated by hundreds to thousands of kilometres, geographic distance explains over 35% of the genetic connectivity in corals^20–22^. On the other hand, along continental coasts such as those of northern Australia^23^, the Caribbean^24^, and eastern Africa^25^, genetic connectivity of corals is largely uncorrelated with geographic distance, likely due to the influence of ocean currents. Biophysical modelling of larval dispersal helps overcome limitations in biological sampling and can clarify complex patterns of genetic connectivity shaped by ocean currents^9,26,27^. For example, the genetic connectivity of a subtidal whelk in California showed no correlation with geographic distance over tens of kilometres, but a biophysical model accounted for nearly 50% of the observed genetic structure ^9^.

For corals in the Nansei Islands, previous biophysical modelling studies^28,29^ have identified the potential for larval transport across more than 1000 km via the Kuroshio Current. However, these studies mainly focused on physical oceanographic processes and did not evaluate the consistency between modelled dispersal and observed genetic connectivity. Previous studies on deep-water corals^30^ and cephalopods^19^ in the Nansei Islands have shown consistent connectivity trends across both biophysical modelling and population genetic analysis. However, these studies were limited to a small part of the archipelago and did not cover the full spatial extent of the Nansei Islands.

Although examples in the Nansei Islands are largely lacking, graph theory-based analysis of connectivity can help to elucidate ecological networks^2,3,31^. Graph theory simplifies the connectivity of habitats, with habitats defined as nodes and the movement pathways connecting them as edges of a network. This approach enables the quantification of ecological network structure, the prioritisation of key corridors, and the evaluation of the importance of each node^2,3,31^. For example, applying graph theory to connectivity based on dispersal modelling of Mediterranean fishes^26^has identified ocean currents that form vital dispersal pathways, as well as islands that serve as important stepping stones. In this study, we utilize graph theory to unravel the complex genetic connectivity patterns previously reported among coral habitats in the Nansei Islands^15–18^.

We investigate ecological networks between coral habitats using a combination of biophysical modelling and population genomic analysis. Our focal species is *Acropora digitifera*, the dominant coral in the archipelago^15^. Biophysical larval dispersal modelling, which releases particles simulating larvae, is employed to quantify larval dispersal networks across all coral habitats in the Nansei Islands. The pelagic larval duration (PLD) is set between 10 and 130 days^32,33^ at 10-day intervals, with particular attention to a 30-day PLD, during which larvae maintain a high settlement capacity^34^. Graph theory-based analysis is then used to identify key stepping stones within these networks. Furthermore, genetic connectivity is analysed based on genome-wide single nucleotide polymorphisms (SNPs) obtained from samples collected throughout the archipelago, and compared against the modelled dispersal. The aim of this study is to elucidate the ecological networks that shape connectivity among coral habitats in the Nansei Islands, thereby informing effective conservation strategies.

## Results

### Larval dispersal networks

**Fig. 2:**
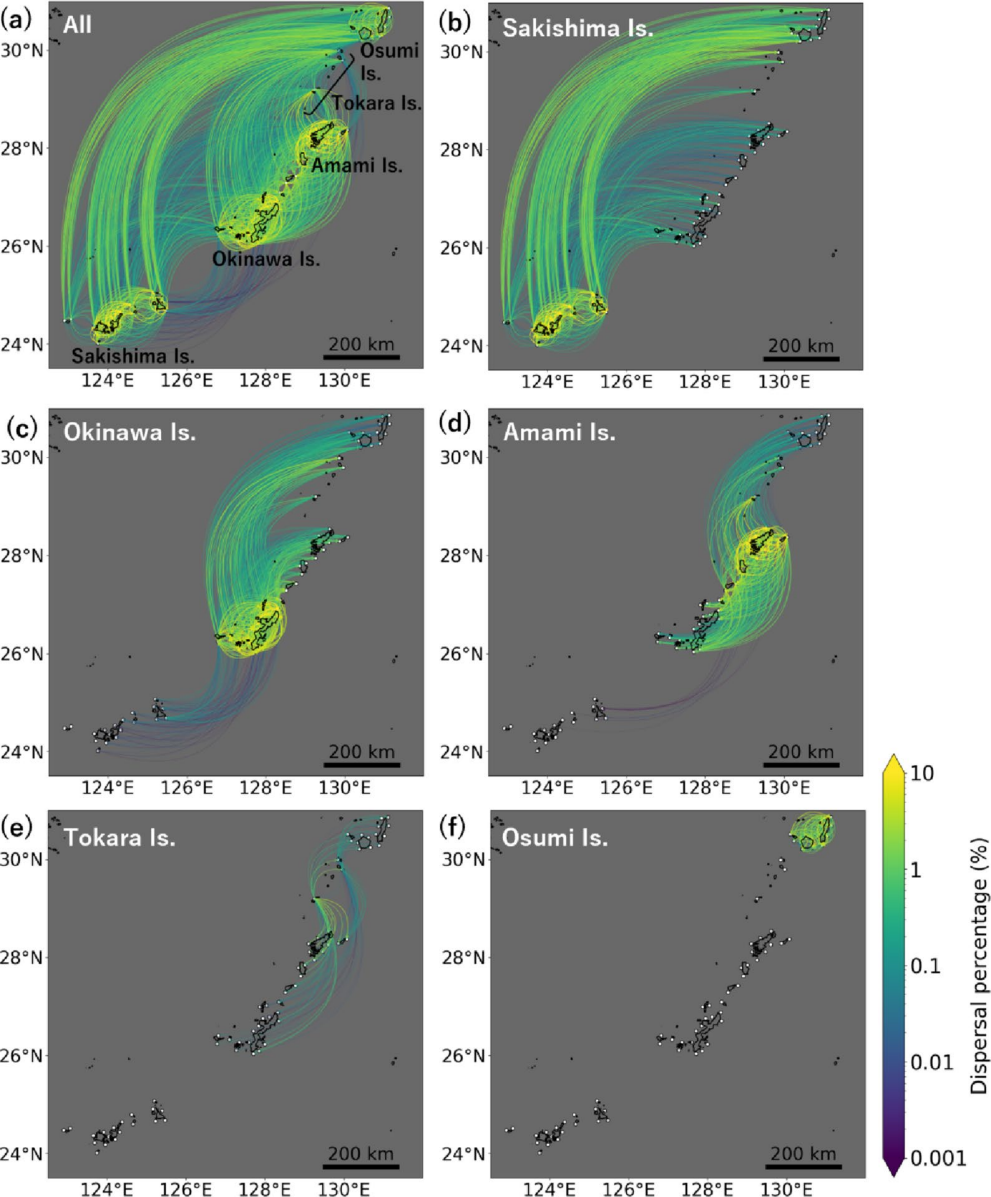
Modelled larval dispersal network with a pelagic larval duration of 30 days. Panels show dispersal with larval sources in (**a**) the entire Nansei Islands, (**b**) the Sakishima Islands, (**c**) the Okinawa Islands, (**d**) the Amami Islands, (**e**) the Tokara Islands, and (**f**) the Osumi Islands. Edge colour represents dispersal percentage between sites, with yellow being higher percentages. Edges are directional, curving clockwise from the start point to the end point. White dots indicate particle release sites.

In the modelled dispersal with PLDs of 10–130 days (Fig. 2, Supplementary Fig. S1), all coral habitats were connected to at least one other habitat, and none were isolated. The larval dispersal network for the 30-day PLD is shown in Fig. 2, and those for the remining PLDs are provided in Supplementary Fig. S1. Unless otherwise noted, subsequent analyses focus on a PLD of 30 days.

The direction of dispersal was predominantly north-easterly along the Kuroshio Current (Fig. 2). Particles released from the southernmost Sakishima Islands dispersed directly to the northernmost Osumi Islands, approximately 800–1100 km away (Fig. 2b). Although less frequent than north-easterly dispersal, south-westerly dispersal also occurred (Figs. 2c–e), potentially influenced by the Kuroshio Counter Current and eddies^28,29^. No particles from the northernmost Osumi Islands dispersed to more southerly islands (Fig. 2f, Supplementary Fig. S2), as the Kuroshio Current, flowing eastward between the Osumi and Tokara Islands, acted as a barrier.

Interestingly, particles from the southernmost Sakishima Islands dispersed more frequently to the northernmost Osumi Islands than to the central Okinawa and Amami Islands (Fig. 2; Table 1). The site-averaged dispersal percentage from the Sakishima Islands to the Osumi Islands (0.77% ± 0.32%; mean ± standard deviation) was six times higher than that to the Okinawa Islands (0.12% ± 0.15%) and 14 times higher than that to the Amami Islands (0.056% ± 0.051%). Furthermore, the Sakishima Islands were the most frequent source of particles reaching the Osumi Islands, despite being the most geographically distant. The second most frequent source was the Okinawa Islands, but their dispersal percentage to the Osumi Islands (0.17% ± 0.081%) was only about one-fifth of that from the Sakishima Islands. Although the Tokara Islands are geographically closest to the Osumi Islands, at approximately 50 km, they exhibited the second-lowest frequency of dispersal to the Osumi Islands (0.10% ± 0.12%). This is likely due to the barrier to dispersal posed by the Kuroshio Current (Supplementary Fig. S2).

**Table 1:** Modelled dispersal percentages (%) between Island groups with a pelagic larval duration of 30 days.

Source\Sink	Sakishima	Okinawa	Amami	Tokara	Osumi
Sakishima	2.9	0.12	0.056	0.82	0.77
Okinawa	0.012	3.5	0.45	0.91	0.17
Amami	0.00020	0.74	4.3	1.5	0.089
Tokara	0	0.068	0.54	1.1	0.10
Osumi	0	0	0	0	1.4

Row names represent particle sources, and column names represent particle sinks. Values are averaged across coastal particle release sites in each island group. Diagonal values represent self-recruitment.

**Fig. 3:**
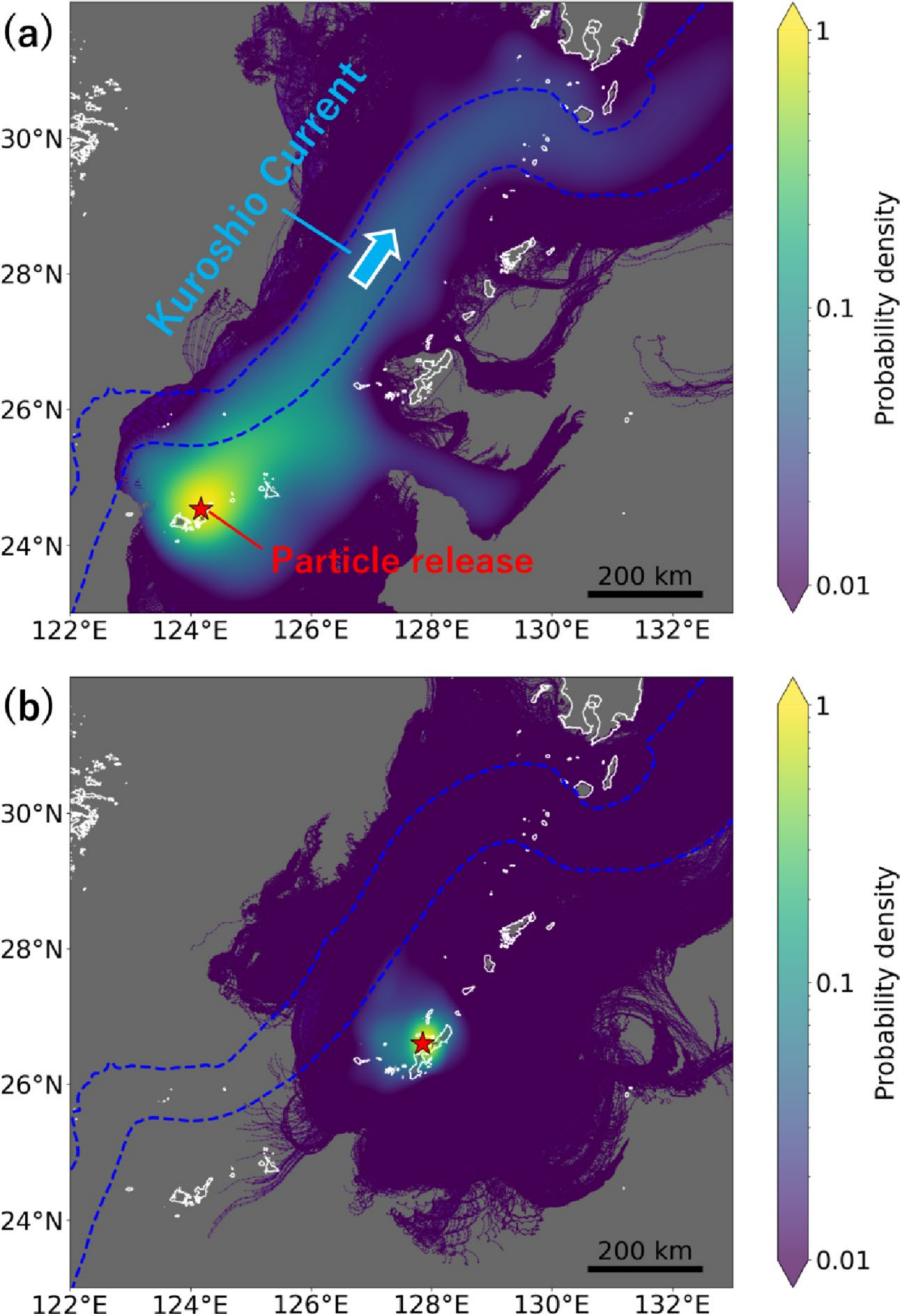
Examples of dispersal pathways with a pelagic larval duration of 30 days. Pathways of 159,600 particles released from (**a**) site Ish-1 in the Sakishima Islands and (**b**) site Oki-5 in the Okinawa Islands are shown. See Fig. 1 for the detailed location of the sites. Colours represent probability densities, with yellow indicating areas where more particles passed through. Red stars mark the particle release sites. The Kuroshio Current region is defined as the area where the 5-year (2019–2023) mean current velocity at 0 m depth during the coral spawning period (May to August) exceeded 4 cm/s and is illustrated by blue dotted lines.

The high frequency of dispersal from the southern to the northern end of the Nansei Islands was attributed to the Kuroshio Current transporting many particles. When defining the Kuroshio Current as a region where the 5-year mean current velocities during the coral spawning period (May to August) exceeded 0.4 m/s (Fig. 1a), 56% of all particles released from the Sakishima Islands entered the Kuroshio Current region (Fig. 3a). These particles bypassed the Okinawa and Amami Islands and dispersed to the Osumi Islands, where the Kuroshio Current directly interacts with the coastline. In contrast, smaller proportions of particles released from the Okinawa and Amami Islands entered the Kuroshio Current region, only 38% and 45%, respectively (Fig. 3b, Supplementary Fig. S2). Compared to the Sakishima Islands, most particles released from the Okinawa and Amami Islands remained near their source sites, indicating more localized dispersal.

**Fig. 4:**
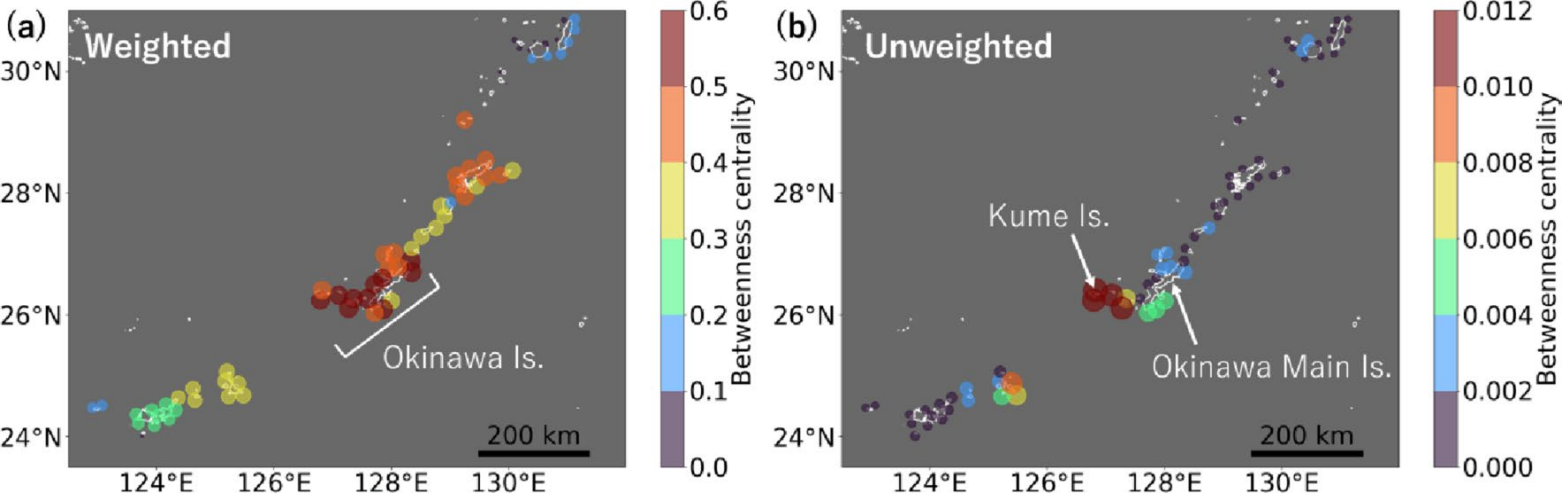
Importance of each site as a stepping stone in the larval dispersal network with a pelagic larval duration of 30 days. The colour and size of the dots indicate betweenness centrality, with the larger and redder dots indicating greater importance as stepping stones. (**a**) shows betweenness centrality weighted by the dispersal percentages between sites, and (**b**) shows unweighted betweenness centrality.

The importance of each node as a stepping stone in the larval dispersal network was quantified using betweenness centrality, which measures the proportion of shortest paths that pass through a given node^35^. Weighted betweenness centrality (Fig. 4a), which emphasises the sites through which more particles passed, was high in the Okinawa Islands (values ≥ 0.5). This is likely due to their central position within the archipelago (Fig. 1), which places them as intermediaries for dispersal between the adjacent Sakishima and Amami Islands. Although the Amami Islands are also centrally located, their betweenness centrality was comparatively lower (values < 0.5), possibly because the Kuroshio Current acts as a barrier to dispersal from the neighbouring Osumi Islands (Fig. 2f).

Unweighted betweenness centrality (Fig. 4b), which highlights sites that connect many other sites regardless of the number of passing particles, was notably high at Kume Island, part of the Okinawa Islands (value > 0.01). Kume Island served as a stepping stone for dispersal to the Sakishima Islands. Particles released from Kume Island reached all 16 sites in the Sakishima Islands, whereas those from the adjacent Okinawa Main Island reached only 2–12 sites, depending on the release location. This difference may be due to Kume Island’s proximity to the Kuroshio Counter Current, which flows southward between the Kuroshio Current and the Okinawa Islands^36^. In contrast, northward dispersal showed little difference between the two islands, with particles from both Kume Island and Okinawa Main Island reaching all 14 sites in the Osumi Islands.

### Comparison between genetic connectivity and modelled dispersal

**Fig. 5:**
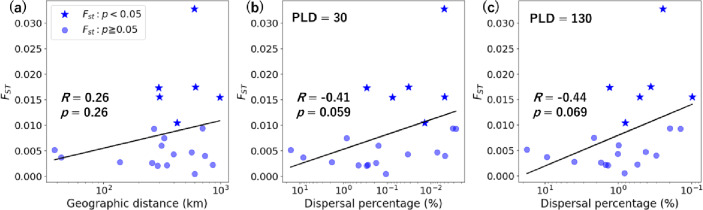
Correlation between genetic differentiation index (*F*_ST_) and: (**a**) geographic distance, (**b**) dispersal percentage with pelagic larval duration (PLD) of 30 days, and (**c**) dispersal percentage with PLD of 130 days. Correlation coefficients (*R*) were obtained using Mantel tests. Stars indicate statistically significant *F*_ST_ values (*p* < 0.05). Dispersal percentages were derived from calculations of ≤ 3 generations of stepping-stone dispersal.

In the population genomic analysis, the genetic differentiation index (*F*_ST_) among all seven sampling sites (Fig. 1) was low (≤ 0.033; Supplementary Tables S1, S2), suggesting gene flow among sites < 1000 km apart. Among all site pairs, genetic differentiation was highest between the Osumi Islands (site Tan-2) and the Okinawa Islands (site Oki-2; *F*_ST_ = 0.033, *p* < 0.05). All other site pairs had *F*_ST_ values below 0.018. The correlation between genetic differentiation and geographic distance was weak (*R* = 0.26, *p* = 0.26; Fig. 5a). When the outlier *F*_ST_ value between sites Tan-2 and Oki-2 was excluded, a negative correlation was observed (*R* = -0.52, *p* = 0.038), even though isolation-by-distance would typically predict a positive correlation^20–22^.

Genetic diversity was similar across all sites (Supplementary Table S3), supporting the possibility of extensive gene flow. Allelic richness ranged from 1.25 (site Oki-2) to 1.36 (site Miy-1). Observed heterozygosity varied from 0.06 (site Oki-2) to 0.08 (sites Iri-5, Miy-1, Ama-4, Ama-7, and Tan-2), while expected heterozygosity ranged from 0.05 (site Oki-2) to 0.08 (site Miy-1). Inbreeding coefficients (*F*_IS_) were negative and significantly different from zero at all sites. The most likely number of genetic populations was *K* = 2, as indicated by both the mean log likelihood and Δ*K*, with individuals from all sites assigned to both clusters (Supplementary Fig. S3).

The correlation between genetic differentiation and dispersal percentages derived from multi-generational stepping-stone dispersal was examined (Fig. 5, Supplementary Table S4). In comparison with geographic distance (*R* = 0.26, *p* = 0.26﻿), dispersal percentages with PLDs of ≥ 20 days showed relatively stronger correlations with genetic differentiation (*R* = -0.44 to -0.31, *p* = 0.059 to 0.18). For a PLD of 30 days, the correlation was *R* = -0.41 (*p* = 0.059; Fig. 5b). The strongest correlation was observed for the longest PLD of 130 days (*R* = -0.44, *p* = 0.069; Fig. 5c). After excluding the outlier *F*_ST_ value between sites Tan-2 and Oki-2, the correlations remained similar (*R* = -0.42, *p* = 0.096 for PLD = 130; Supplementary Table S5).

## Discussion

All coral habitats in the Nansei Islands formed a single, interconnected larval dispersal network (Fig. 2). Particles released from the southernmost Sakishima Islands dispersed to the northernmost Osumi Islands more than six times as frequently as to the central islands (Fig. 2; Table 1), primarily due to transport by the Kuroshio Current (Fig. 3). Low genetic differentiation and similar genetic diversity between sites (Supplementary Tables S1, S3) indicated potential gene flow across the archipelago, supporting a single larval dispersal network. Genetic differentiation showed a relatively stronger correlation with modelled dispersal than with geographic distance (Fig. 5). These findings suggest that coral habitats in the Nansei Islands are connected by a single larval dispersal network, including dispersal pathways that facilitate long-distance connectivity via the Kuroshio Current.

In the modelled dispersal, the Kuroshio Current created a pathway connecting the islands at both ends of the archipelago while bypassing the central islands (Fig. 2). We refer to this pathway as the ‘Kuroshio Corridor’. This corridor, together with the resulting larval dispersal network, helps to explain the complex genetic connectivity in the region^15–18^. Previous population genetics studies on corals suggested that gene flow did not simply follow the linear distribution of islands, with strong gene flow reported from the southern to northern ends of the Nansei Islands^15,18^. This pattern is consistent with our modelled dispersal network (Fig. 2b). Previous research^15,18^did not clarify whether this long-distance genetic connectivity occurred through direct dispersal or via stepping-stone routes. However, our modelling shows that direct dispersal from the southern to the northern islands could occur at a relatively high frequency. Population genetics studies of corals^37,38^ and fishes^39^ have shown that genetic lineages are separated north and south of the Osumi Islands, likely because the Kuroshio Current flowing south of the Osumi Islands acted as a dispersal barrier^38,39^. In this study, no dispersal occurred from the Osumi Islands to more southerly islands (Fig. 2f), supporting these earlier interpretations. Collectively, larval dispersal networks—including the Kuroshio Corridor—provide a basis for better understanding of the complex genetic connectivity patterns around the Nansei Islands.

The strong connectivity between remote islands facilitated by the Kuroshio Corridor contrasts with findings from other archipelagos, where coral connectivity tends to decline with increasing geographic distance^20–22^. Genetic differentiation of *A. digitifera* along a 4000-km-long archipelago in Micronesia is primarily determined by geographic distance (*R*^2^ = 0.62, *p* = 0.007)^20^. Similarly, over 35% of the genetic differentiation of corals across the 2600-km-long Hawaiian Islands^21^and the 600-km-long Lesser Antilles^22^is explained by geographic distance. These archipelagos, like the Nansei Islands, form near-linear island chains spanning hundreds to thousands of kilometres. In contrast, this study found that genetic differentiation had a weak correlation with geographic distance (*R* = 0.26, *p* = 0.26) but a relatively stronger correlation with modelled dispersal (*R* = -0.44, *p* = 0.069; Fig. 5). This suggests that the Nansei Islands may be unique, as ocean currents bypassing the central region create inter-island connectivity that depends less on geographical continuity than in other archipelagos^20–22^. Thus, simple extrapolation of findings from other archipelagos may not be sufficient to fully understand the connectivity of corals in the Nansei Islands.

Our population genetic findings may contribute to understanding gene flow shaping evolutionary processes among populations, rather than a demographically meaningful influx of numerous individuals. The strongest correlation between genetic differentiation and modelled dispersal was observed for the longest PLD of 130 days (Fig. 5, Supplementary Table S4). Previous laboratory experiments^33^ have shown that the survival rate of *Acropora* larvae decreases to ~ 1% by 130 days after spawning. Accordingly, our population genomic analysis may have detected genetic connectivity resulting from rare long-distance dispersal events involving a small number of larvae. Indeed, genetic connectivity can be maintained by as few as several migrants per generation^6,7^. Meanwhile, at the 30-day PLD—the primary focus of this study and a period during which larvae maintain high settlement capacity^32,34^—the survival rate of *Acropora* larvae remains relatively high (≥ 10%)^32,33^. Modelled dispersal networks for longer PLDs therefore appear to primarily offer insights into evolutionary processes, whereas those for PLDs ≤ 30 days may additionally inform demographic processes.

Our modelling focuses on the physical potential for dispersal and does not account for post-dispersal barriers to successful reproduction^40,41^. Individuals that disperse to a new environment tend to experience higher mortality compared to those originating from that area^40,41^. The northern end of the Nansei Islands marks the northern limit of coral reef distribution in the western Pacific Ocean^13^and may present a harsh environment for larvae dispersing from the south. For example, mean water temperatures around the northern end of the archipelago are approximately 3 °C lower than those around the southern end (Supplementary Fig. S4). One future challenge will be to incorporate post-dispersal barriers, such as mortality, into biophysical models, which may lead to a stronger correlation between genetic differentiation and modelled dispersal.

Population genetics studies often require the estimation of complex sink-source relationships based on limited sampling locations^42^, but our larval dispersal networks can help to overcome this limitation. A previous study^16^analysed gene flow using samples collected from islands south of the Okinawa Islands and suggested that the Sakishima Islands were an important larval source to the Okinawa Islands. However, our modelling indicated the main larval source to the Okinawa Islands was not the Sakishima Islands but the Amami Islands, which lie north of the Okinawa Islands and were not sampled in the previous study^16^. Modelled dispersal to the Okinawa Islands occurred six times more frequently from the Amami Islands (dispersal percentage = 0.74% ± 0.62%) than from the Sakishima Islands (dispersal percentage = 0.12% ± 0.15%; Fig. 2). Our larval dispersal network across the Nansei Islands may help to fill connectivity gaps at sites where biological sampling has not yet been conducted.

The larval dispersal networks provide insights for the conservation of coral connectivity. Identifying and conserving larval source populations is crucial for supporting the recovery of local populations following disturbances^3,4^. Our larval dispersal networks can contribute to clarifying the overall structure of complex sink-source relationships within the Nansei Islands, particularly at the inter-island scale. Maintaining regional connectivity also requires conserving key stepping stones within ecological networks^2,31^. Our analysis indicated that Kume Island served as a relatively important stepping stone, facilitating larval exchange among many sites (Fig. 4b). Although many coastal areas in the Nansei Islands are designated as national parks, Kume Island is not included (https://www.env.go.jp/en/nature/nps/park/parks/index.html, accessed 1st May 2025). Coral habitats on Kume Island have experienced declines in live coral cover and diversity due to bleaching and other stressors^43^. Given their poor condition, conservation attention may be warranted for coral habitats on Kume Island—not only to support local populations but also to help maintain regional connectivity. Our findings suggest that understanding the larval dispersal networks linked by the Kuroshio Corridor is crucial for developing effective conservation plans for coral habitats.

## Methods

### Study area

The biophysical modelling covered all coral habitats in the Nansei Islands that were surveyed by the Japanese Ministry of the Environment (http://gis.biodic.go.jp/webgis/index.html, accessed 1st October 2024; Fig. 1). The Ministry conducted assessments of coral distribution between 1993 and 2021, using satellite data alongside field observations. In this study, coral habitats were defined as areas where corals covered at least 5% of the seabed.

### Biophysical modelling

#### Larval ecology settings

Members of the genus *Acropora* are broadcast spawners that release buoyant sperm and eggs, which are fertilised in the water column to form larvae. Previous laboratory experiments have shown that *A. digitifera* larvae can survive for at least 54 days^32^, and larvae of other *Acropora* species have been reported to survive for over 130 days^33^. Given that genetic connectivity can be maintained by very small number of migrants that survived over the long term^6,7^, we conducted simulations with PLDs at 10-day intervals ranging from 10 to 130 days. *Acropora* larvae can maintain high settlement capacity for up to approximately 30 days after spawning^34^. Thus, we primarily focused on a PLD of 30 days. The age-dependent decline in larval survival rate^32^ was not incorporated into the model.

Laboratory experiments involving eight *Acropora* species have reported a pre-competency period (i.e., the duration from spawning until larvae become capable of settlement) of approximately 4–6 days^34^. In this study, we set the pre-competency period to 4 days.

Based on 493 spawning records of *Acropora* species from the Nansei Islands^44^, the spawning window was set to 18:00–24:00 h between May and August. We further constrained this window to within ± 6 days of the full moon, a period during which 89% of recorded spawning events occurred^44^. Full-moon dates were obtained from the Japan Meteorological Agency’s records for Naha City, Okinawa Main Island (https://www.data.jma.go.jp/kaiyou/db/tide/suisan/suisan.php?stn=NH, accessed 11 January 2026).

### Larval dispersal simulation

Larval dispersal simulations were conducted using Lagrangian particle tracking with the Python package ‘Parcels’^45^. Larvae were assumed to be passively transported by horizontal currents. To avoid introducing substantial complexity into the model, we did not incorporate larval vertical movement^46^, wind-driven transport of slicks formed by eggs and larvae^47^, or sub-grid scale turbulence. Note that these processes may modify the flow fields experienced by larvae and thereby affect dispersal outcomes.

Current data were obtained from the ocean model JCOPE-T^48^, which covers the waters surrounding Japan. JCOPE-T has a horizontal resolution of 1/36° (≈ 3 km), a vertical resolution of 46 layers in generalised σ coordinates, and a time interval of one hour. To enhance accuracy, satellite observations of sea surface height and temperature, as well as in situ measurements of temperature and salinity profiles, are assimilated into the model. As JCOPE-T accurately reproduces the path of the Kuroshio Current^48^, it was considered a suitable model for the study area. The annual mean flow derived from JCOPE-T showed no major inter-annual variability throughout the 5-year calculation period (2019–2023; Supplementary Fig. S5).

A total of 68 particle release sites were established within the coral habitats at 15-km intervals (Fig. 1b–f). Particles were released at 3-hour intervals during the spawning window (described above) from 2019 to 2023. At each interval, two hundred particles were released per site and were initially placed randomly within a 3 × 3 km^2^ area centred on the site. This resulted in 159,600 particles being released per site, and a total of 10,852,800 particles across all 68 sites. The depth of dispersal was set to 0 m. The simulation domain was 120°–135°E and 21°–34°N, and particle tracking was terminated once a particle exited this domain.

The dispersal percentage was defined as the proportion of particles released from site $\:A$ that reached site $\:B$, calculated as:

1 $$\:P=100\times\:\frac{{N}_{A\to\:B}}{{N}_{all}},$$

where $\:P$ is the dispersal percentage (%), $\:{N}_{A\to\:B}$ is the number of particles released from site $\:A$ and passing through a 3 × 3 km^2^ area centred on site $\:B$, and $\:{N}_{all}$ is the total number of particles released from site $\:A$. Only trajectories occurring after the 4-day pre-competency period (described above) were included in the calculation. All particles that passed through the focal site were treated as having dispersed successfully; neither a decline in settling capacity^34^ nor post-dispersal mortality^40,41^ was considered. The dispersal percentage from Islands $\:A$ to Islands $\:B$ was then obtained by averaging the dispersal percentages from all coastal particle release sites in Islands $\:A$ to all coastal particle release sites in Islands $\:B$.

To visualise major dispersal pathways, the probability density of the particle distribution was obtained using kernel density estimation with the ‘kern-smooth’ package in Python (https://github.com/AlexanderButyaev/kern_smooth, accessed 1st May 2025). A Gaussian kernel function was used for weighting.

### Network analysis

Directed graphs showing larval dispersal networks were created using the ‘NetworkX’ package in Python (https://github.com/networkx/networkx, accessed 1st May 2025). Particle release sites were treated as nodes, and dispersal percentages between sites were used as edge weights. The network included only direct dispersal and did not incorporate multi-generational stepping-stone dispersal, which is described below.

### Betweenness centrality

Betweenness centrality was calculated using the ‘betweenness_centrality’ function in NetworkX. The betweenness centralities were normalized by $\:1/\left(\left(n-1\right)\left(n-2\right)\right),$ where $\:n$ is the number of nodes in the network. As in^31^, two types of betweenness centrality were calculated: weighted and unweighted. The dispersal percentage $\:P$ was converted into a distance measure, $\:\mathrm{log}(1/P)$, in accordance with^35^, and used as weight indicating the distance between nodes. Weighted betweenness centrality emphasises nodes that are traversed by more particles. On the other hand, unweighted betweenness centrality becomes high for nodes that lie on the shortest paths between many others, regardless of the number of particles passing through them^31^.

### *Multi-generational stepping-stone dispersal*

For comparison with genetic differentiation derived from population genomic analysis, dispersal percentages resulting from stepping-stone dispersal over multiple generations were obtained following the method outlined in^26^. Using the Floyd-Warshall algorithm to solve the shortest path problem between all node pairs in a weighted directed graph, the stepping-stone dispersal percentage $\:{P}_{1,n}$ from the first node to the $\:n$-th node was calculated in Python as follows:

2 $$\:{P}_{1,\:n}={P}_{1,\:2}\times\:{P}_{2,\:3}\times\:\cdots\:\times\:{P}_{n-1,n}.$$

Here, the dispersal percentage $\:P$ is converted to $\:\mathrm{log}(1/P)$ as distance measures, following^35^. Therefore, the relationship holds:

3 $$\:\mathrm{log}\left(\frac{1}{{P}_{1,n}}\right)=\mathrm{log}\left(\frac{1}{{P}_{\mathrm{1,2}}}\right)+\mathrm{log}\left(\frac{1}{{P}_{\mathrm{2,3}}}\right)+\cdots\:+\mathrm{log}\left(\frac{1}{{P}_{n-1,n}}\right).$$

The path with the smallest $\:\mathrm{log}\left(1/{P}_{1,n}\right)$ was identified as the shortest path. As in^26^, stepping-stone dispersal through $\:n$-th nodes was considered to take $\:n-1$ generations. Since dispersal percentage is a vector quantity, it has different values between sites, depending on which of the two sites is the source or sink of the dispersal. In this study, the larger value was defined as the dispersal percentage between the two sites and compared with genetic differentiation.

For all biological sampling sites to be connected, stepping-stone dispersal required four generations at a PLD of 10 days, three generations at PLDs of 20 and 30 days, two generations at PLDs of 40 and 50 days, and one generation at PLDs of ≥ 60 days.

### Population genomic analysis

For population genomic analysis of *A. digitifera*, seven sites (Fig. 1, Supplementary Table S6) were selected from previously reported samples^15^, with 8–16 samples per site. These sites span the entire Nansei Islands, and were therefore considered suitable for examining broad-scale population genetic structure. Genomic DNA was extracted by previous study^15^.

Double-digest restriction-site-associated DNA sequencing (ddRAD-seq) was applied to samples from each site^49^. We prepared a RAD-seq library using the method described in^50^, with EcoRI and BglII enzymes, and performed size selection at around 320 bp. The final ddRAD-seq libraries were sequenced using an Illumina HiSeq 2500 sequencer (Illumina, San Diego, CA, USA) with 100 bp paired-end reads.

The raw reads were first aligned to the reference genome of *A. digitifera*^51^ by using Bowtie2 v2.3.5.1^52^ with the default setting. The outputs of Bowtie were converted and sorted to BAM files, as suggested by^53^. The aligned reads were analysed with the ref_map.pl pipeline in Stacks v2.66^54^ at the default setting to identify SNPs. The populations module of Stacks was run with the following parameters: the locus must be present in at least 80% of individuals across populations (populations: -R) and present in all populations (-p), minor allele frequency was set up to 0.02 (--min-maf), and the maximum observed heterozygosity was set to 0.5 (--max-obs-het). The SNP dataset was filtered by using VCFtools^55^ with the following options: -min-alleles 2 --max-alleles 2 --max-missing 0.8 --maf 0.02. We removed individuals with more than 20% missing data. We further filtered the SNP dataset by using PLINK2^56^ to exclude SNPs showing deviation from Hardy–Weinberg equilibrium (--hwe 0.001), and the SNP dataset was thinned based on linkage disequilibrium (--indep-pairwise 50 5 0.2). We identified SNPs possibly affected by natural selection by using OutFLANK v0.2^57^, and one locus was identified as an outlier and consequently removed from the SNP dataset. Finally, we used the script vcf_clone_detect.py (https://github.com/pimbongaerts/radseq/blob/master/vcf_clone_detect.py) to identify clones in the SNP dataset based on allelic similarity between all individuals. We identified clones as individuals with > 97% pairwise similarity and kept one sample per clone. The final SNP dataset consisted of 520 SNPs from 71 individuals.

Pairwise *F*_ST_ values between populations were calculated by using the ‘stamppFst’ function from the R package ‘StAMPP’^58^ with 10,000 bootstrap replications, and *p*-values were adjusted for multiple comparisons by using the Benjamini-Hochberg procedure^59^. The allelic richness, observed and expected heterozygosity, and *F*_IS_ were calculated using the ‘divBasic’ function of the R package ‘diveRsity’^60^, with 1000 bootstrap replications to compute 95% confidence intervals.

A Bayesian model-based clustering analysis was performed using Structure v2.3.4^61^ to infer the population structure in the study area. All runs were performed in parallel using StrAuto v1.0^62^. For each value of *K* = 1–9, ten independent replicates were performed using 10,000,000 MCMC iterations following a burn-in of 1,000,000 iterations. The optimal number of genetic clusters was determined using the mean log likelihood and the Δ*K* method of^63^, as implemented in Structure Harvester^64^.

The correlations between *F*_ST_ and both dispersal percentage and geographic distance were assessed using the Mantel tests. The analysis employed the Python package ‘mantel’ (https://github.com/jwcarr/mantel, accessed 1st December 2025). The permutation number was set to 10,000. Geographic distance between sampling sites was calculated using Euclidean distance.

## Supplementary Information

Below is the link to the electronic supplementary material.

Supplementary Material 1

## Data Availability

The data that support the findings of this study are openly available in Zenodo at https://zenodo.org/records/18332882.
