# Supplementary material for: Kuroshio Corridor: larval dispersal networks explain geographically independent connectivity among coral habitats in Japan
Source: Sci Rep. 2026 Feb 20;16:11757. doi: 10.1038/s41598-026-40448-z (PMC13066514; doi:10.1038/s41598-026-40448-z)
Supplement: Supplementary file 1 — Supplementary Material 1 [file 41598_2026_40448_MOESM1_ESM.docx]

**Supplementary information** for

*Kuroshio Corridor: Larval dispersal networks explain geographically independent connectivity among coral habitats in Japan*

**Tables**

Table S1. Genetic differentiation index (*F*_ST_) between sampling sites. Values with *p* < 0.05 are shown in bold. See Fig. 1 for the location of the sites.

| Site | Iri-5 | Miy-1 | Ker-1 | Oki-2 | Ama-4 | Ama-7 |
| --- | --- | --- | --- | --- | --- | --- |
| Miy-1 | 0.00278 |  |  |  |  |  |
| Ker-1 | 0.00432 | 0.00257 |  |  |  |  |
| Oki-2 | **0.0104** | 0.00206 | 0.00368 |  |  |  |
| Ama-4 | 0.00936 | 0.00469 | 0.00601 | **0.0173** |  |  |
| Ama-7 | 0.00397 | 0.000484 | 0.00212 | 0.00749 | 0.00512 |  |
| Tan-2 | **0.0155** | 0.00221 | **0.0175** | **0.0328** | **0.0155** | 0.00928 |

Table S2. *p*-values for genetic differentiation index (*F*_ST_; Tables S1) between sampling sites. See Fig. 1 for the location of the sites.

| Site | Iri-5 | Miy-1 | Ker-1 | Oki-2 | Ama-4 | Ama-7 |
| --- | --- | --- | --- | --- | --- | --- |
| Miy-1 | 0.270 |  |  |  |  |  |
| Ker-1 | 0.192 | 0.264 |  |  |  |  |
| Oki-2 | 0.0284 | 0.320 | 0.259 |  |  |  |
| Ama-4 | 0.0720 | 0.167 | 0.167 | 0.00210 |  |  |
| Ama-7 | 0.273 | 0.467 | 0.374 | 0.167 | 0.270 |  |
| Tan-2 | 0.0349 | 0.401 | 0.0224 | 0.00 | 0.0368 | 0.167 |

Table S3. Allelic richness, observed (*H*_O_) and expected (*H*_E_) heterozygosity, and inbreeding coefficient (*F*_IS_) at each site. See Fig. 1 for the location of the sites.

| Site | Population size | Allelic richness | *H*_O_ | *H*_E_ | *F*_IS_ |
| --- | --- | --- | --- | --- | --- |
| Miy-1 | 15 | 1.36 | 0.08 | 0.08 | -0.0793 |
| Iri-5 | 10 | 1.32 | 0.08 | 0.07 | -0.107 |
| Ama-7 | 7 | 1.30 | 0.08 | 0.07 | -0.125 |
| Ker-1 | 14 | 1.32 | 0.07 | 0.07 | -0.0831 |
| Ama-4 | 9 | 1.32 | 0.08 | 0.07 | -0.116 |
| Oki-1 | 14 | 1.25 | 0.06 | 0.05 | -0.100 |
| Tan-2 | 6 | 1.27 | 0.08 | 0.07 | -0.131 |

Table S4. Correlation between genetic differentiation index (*F*_ST_) and dispersal percentages for each pelagic larval duration (PLD). Correlation coefficients (*R*) were obtained using Mantel tests. Dispersal percentages were derived from calculations of ≤4 generations of stepping-stone dispersal.

| PLD | 10 | 20 | 30 | 40 | 50 | 60 | 70 | 80 | 90 | 100 | 110 | 120 | 130 |
| --- | --- | --- | --- | --- | --- | --- | --- | --- | --- | --- | --- | --- | --- |
| *R* | -0.17 | -0.31 | -0.41 | -0.34 | -0.40 | -0.39 | -0.40 | -0.41 | -0.42 | -0.43 | -0.43 | -0.44 | -0.44 |
| *p* | 0.53 | 0.18 | 0.059 | 0.15 | 0.086 | 0.10 | 0.097 | 0.087 | 0.087 | 0.077 | 0.074 | 0.073 | 0.069 |

Table S5. Correlation between genetic differentiation index (*F*_ST_) and dispersal percentages for each pelagic larval duration (PLD), when the outlier *F*_ST_ value between sites Tan-2 and Oki-2 was excluded. Correlation coefficients (*R*) were obtained using Mantel tests. Dispersal percentages were derived from calculations of ≤4 generations of stepping-stone dispersal.

| PLD | 10 | 20 | 30 | 40 | 50 | 60 | 70 | 80 | 90 | 100 | 110 | 120 | 130 |
| --- | --- | --- | --- | --- | --- | --- | --- | --- | --- | --- | --- | --- | --- |
| *R* | -0.12 | -0.26 | -0.52 | -0.33 | -0.38 | -0.38 | -0.40 | -0.40 | -0.40 | -0.41 | -0.41 | -0.42 | -0.42 |
| *p* | 0.66 | 0.27 | 0.038 | 0.17 | 0.12 | 0.12 | 0.11 | 0.10 | 0.11 | 0.10 | 0.10 | 0.098 | 0.096 |

Table S6. Location of biological sampling sites.

| Site name | Location |
| --- | --- |
| Iri-5 | Kuroshima Is. |
| Miy-1 | Irabujima Is. |
| Ker-1 | Majanohama, Akajima Is. |
| Oki-1 | Ohdo, Okinawajima Is. |
| Ama-4 | Naon, Amamiohshima Is. |
| Ama-7 | Kusunoki, Amamiohshima Is. |
| Tan-2 | Sumiyoshi, Tanegashima Is. |

**Figures**


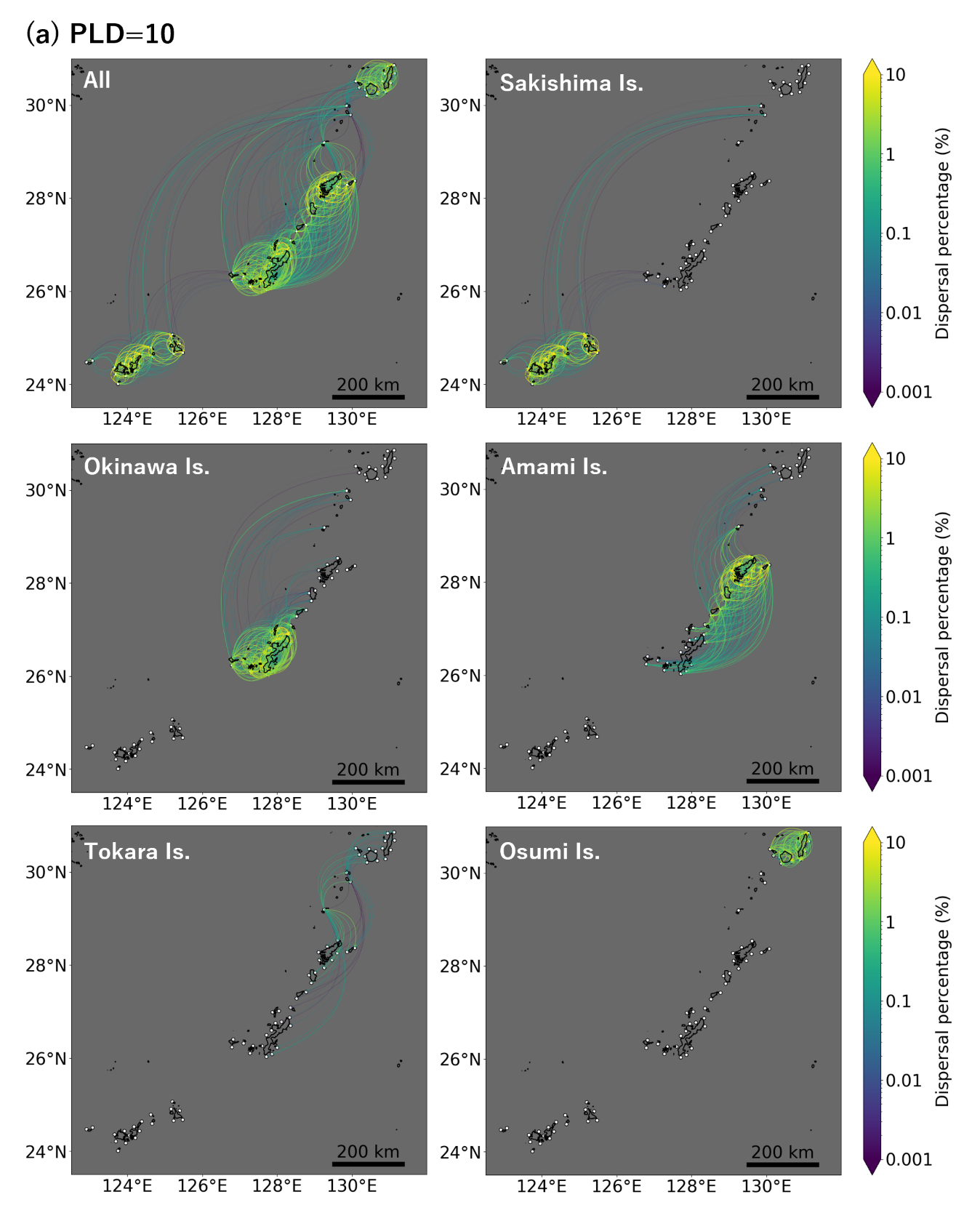


Fig. S1. Modelled larval dispersal network with pelagic larval durations (PLDs) of (a) 10, (b) 20, (c) 60, and (d) 130 days. Panels show dispersal with larval sources in the entire Nansei Islands (top left), the Sakishima Islands (top right), the Okinawa Islands (middle left), the Amami Islands (middle right), the Tokara Islands (bottom left), and the Osumi Islands (bottom right). Edge colour represents dispersal percentage between sites, with yellow being higher percentages. Edges are directional, curving clockwise from the start point to the end point. White dots indicate particle release sites.


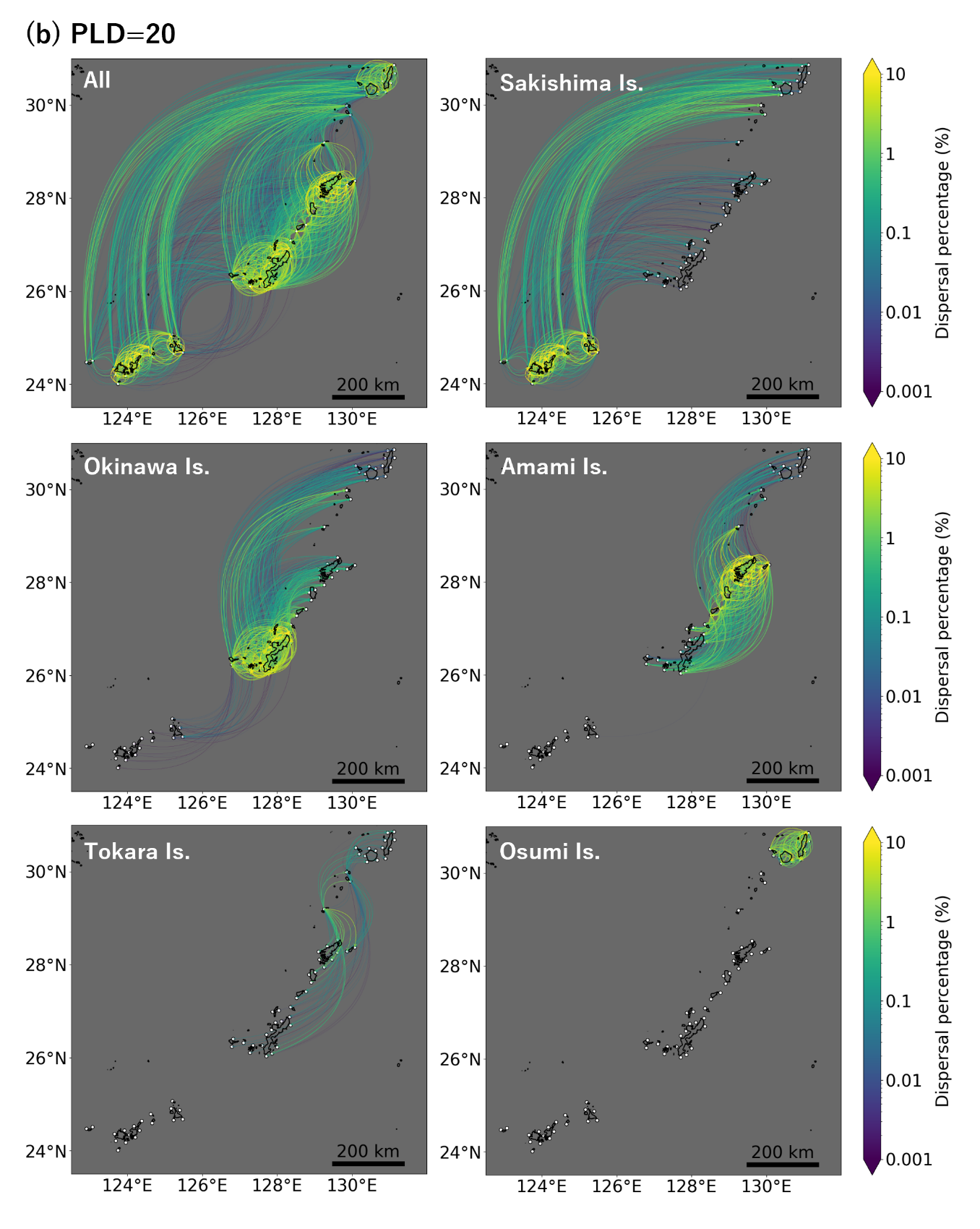


Fig. S1 Continued.


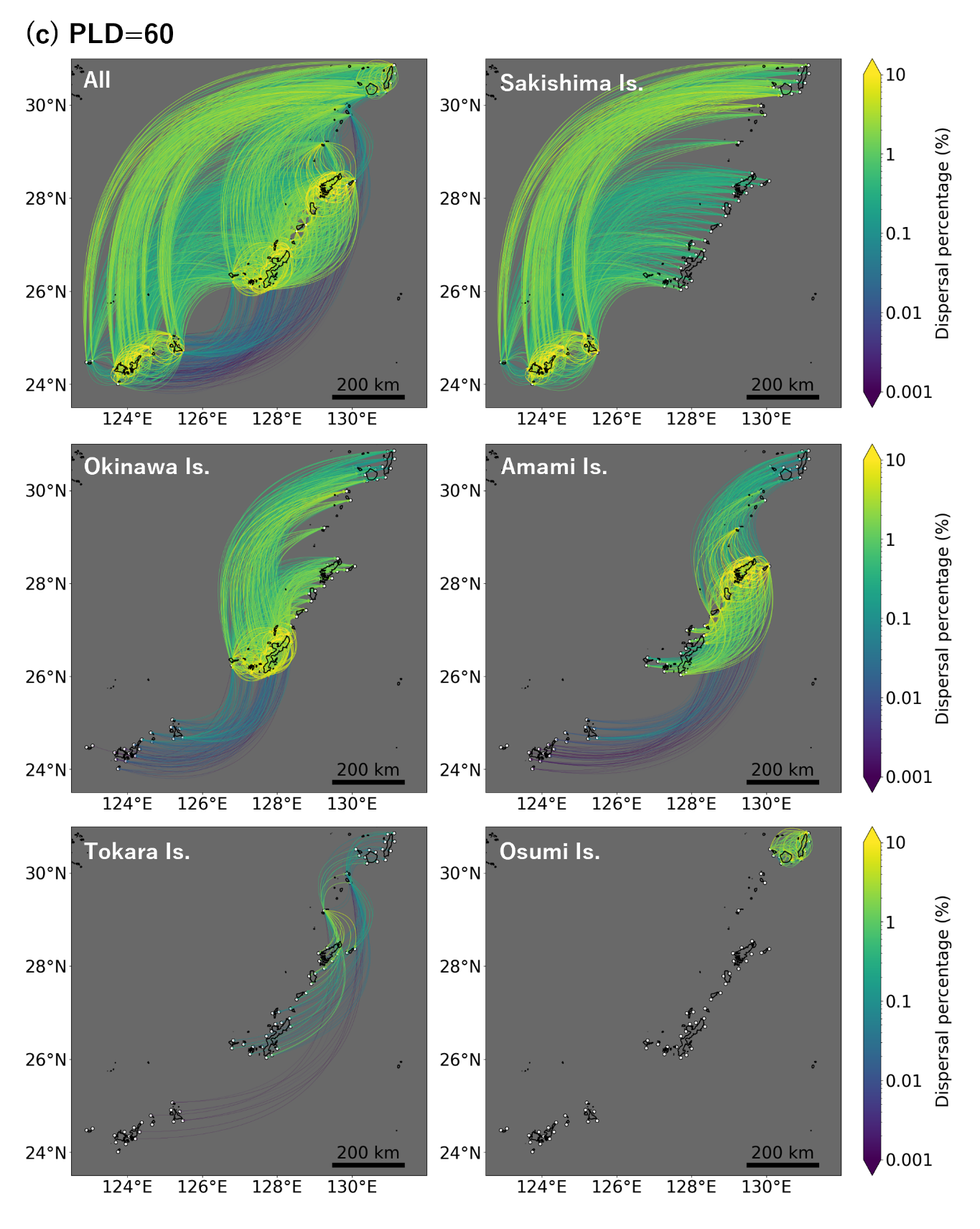


Fig. S1 Continued.


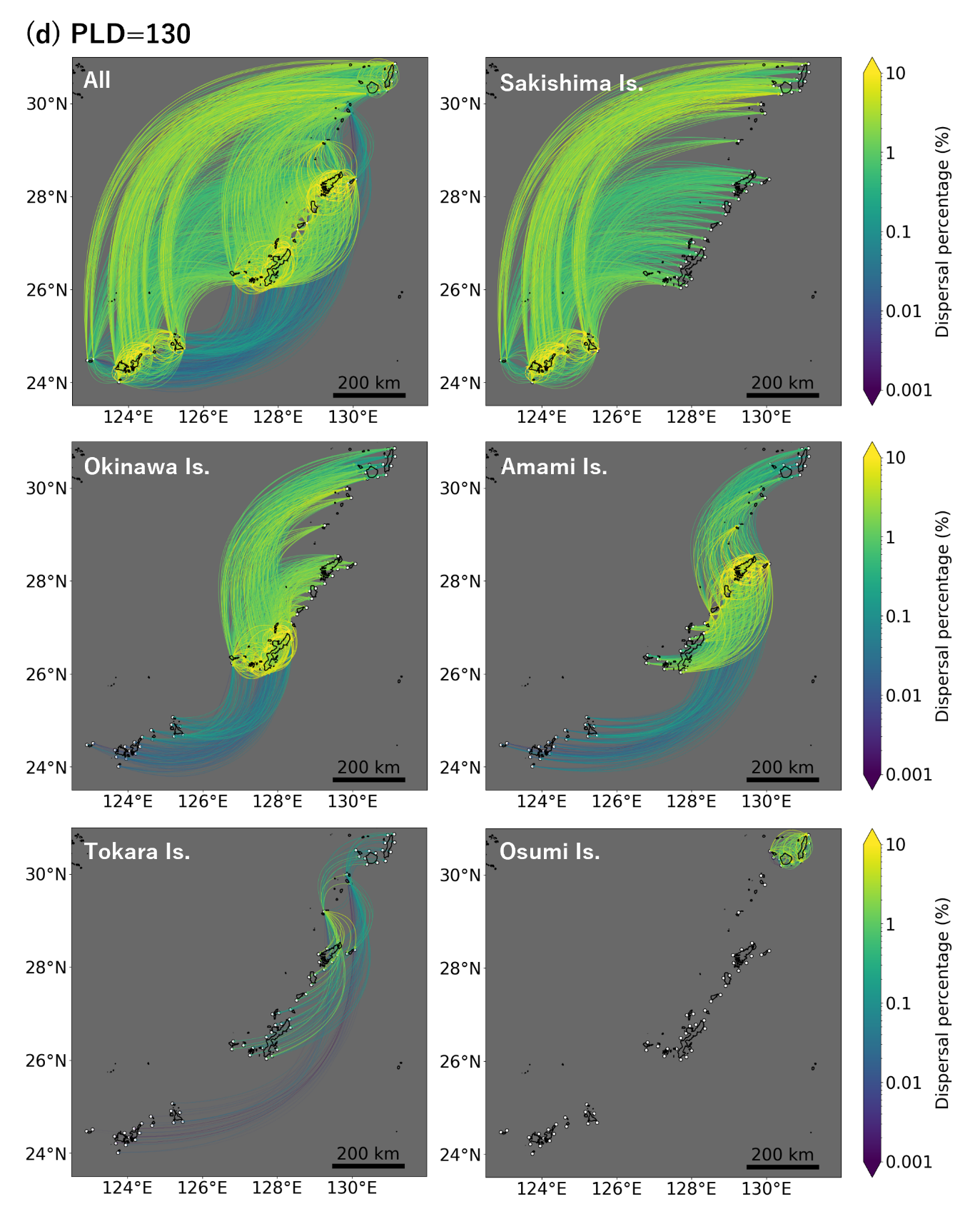


Fig. S1 Continued.


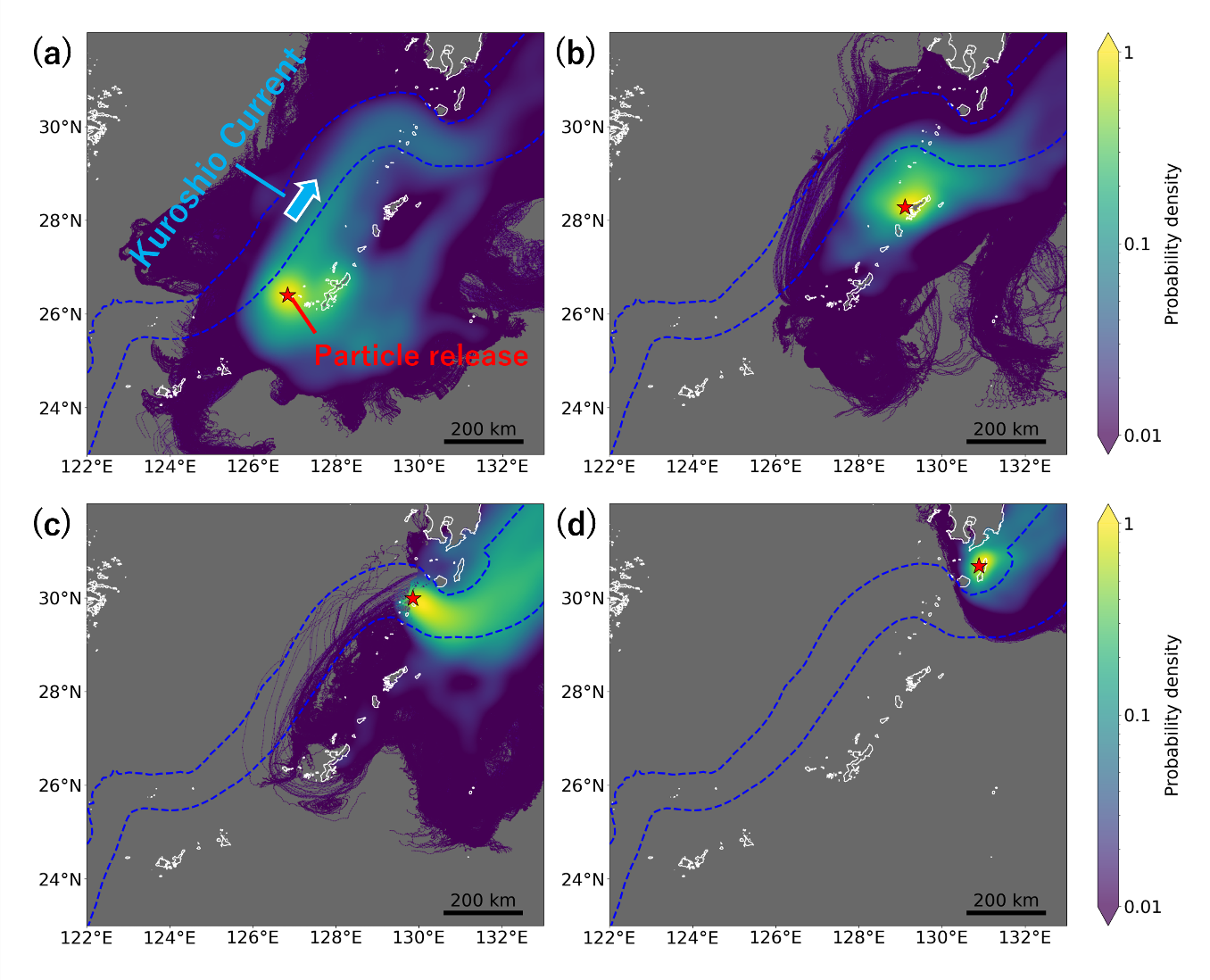


Fig. S2. Examples of dispersal pathways with a pelagic larval duration of 30 days. Pathways of 159,600 particles released from (a) site Kum-2 on Kume Island, (b) site Am-1 in the Amami Islands, (c) site Toka-2 in the Tokara Islands, and (d) site Tan-2 in the Osumi Islands are shown. See Fig. 1 for the detailed location of the sites. Colours represent probability densities, with yellow indicating areas where more particles passed through. Red stars mark the particle release sites. The Kuroshio Current region is defined as the area where the 5-year (2019–2023) mean current velocity at 0 m depth during the coral spawning period (May to August) exceeded 4 cm/s and is illustrated by blue dotted lines.


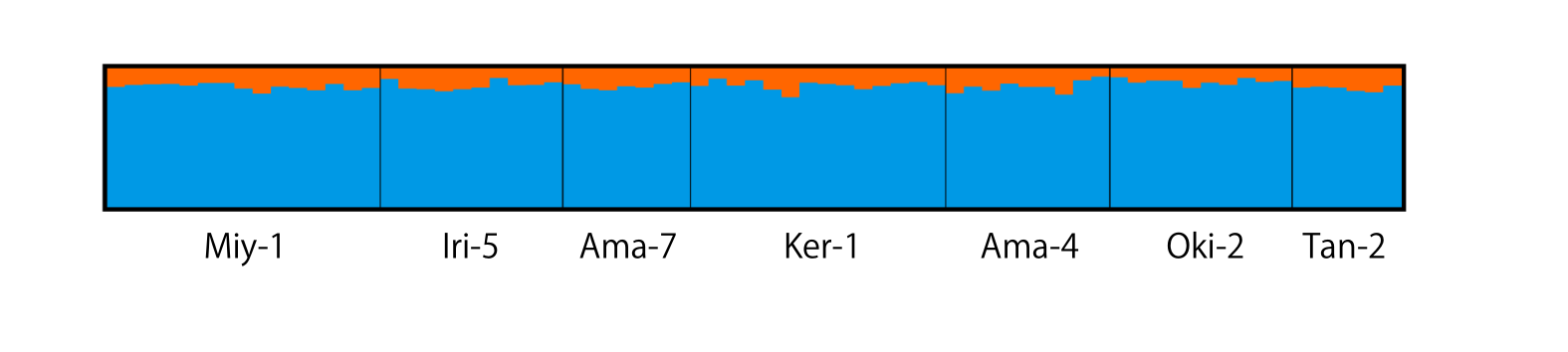


Fig. S3. Structure results for *K* = 2, with individuals plotted on the x-axis and assignment probabilities on the y-axis.


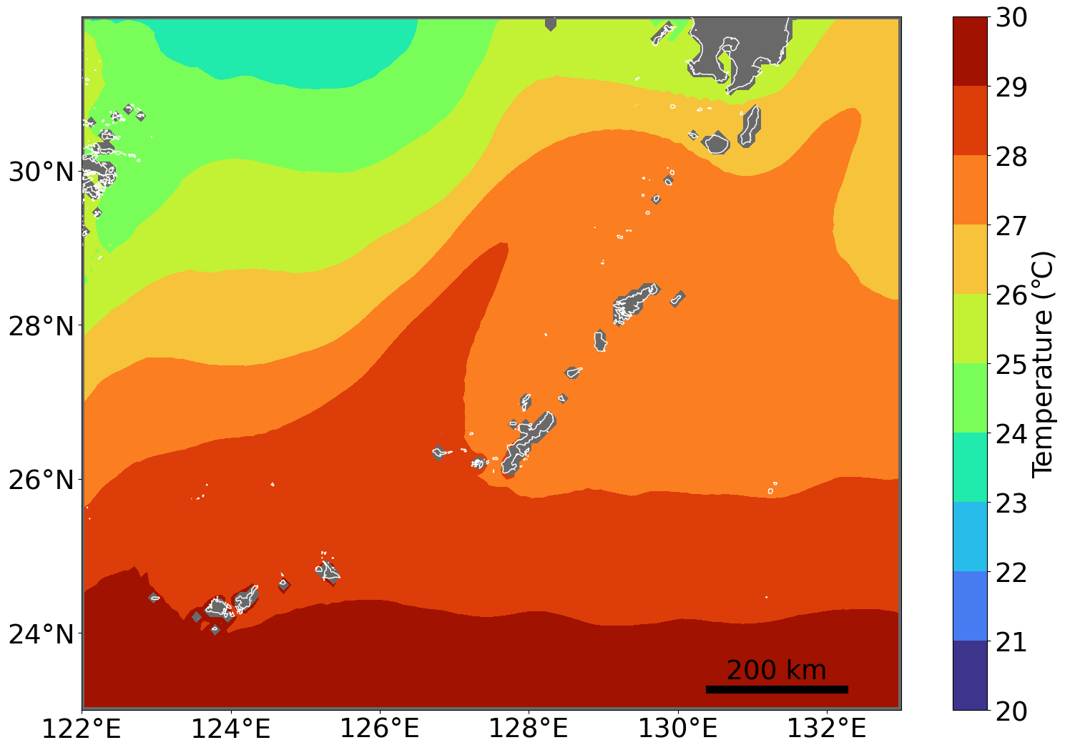


Fig. S4. Five-year (2019–2023) mean water temperature at 0 m depth during the coral spawning period (May to August). Data were obtained from JCOPE2M (Miyazawa et al. 2017; Miyazawa et al. 2018), a low-resolution version (1/12° horizontal resolution) of the ocean model JCOPE-T.


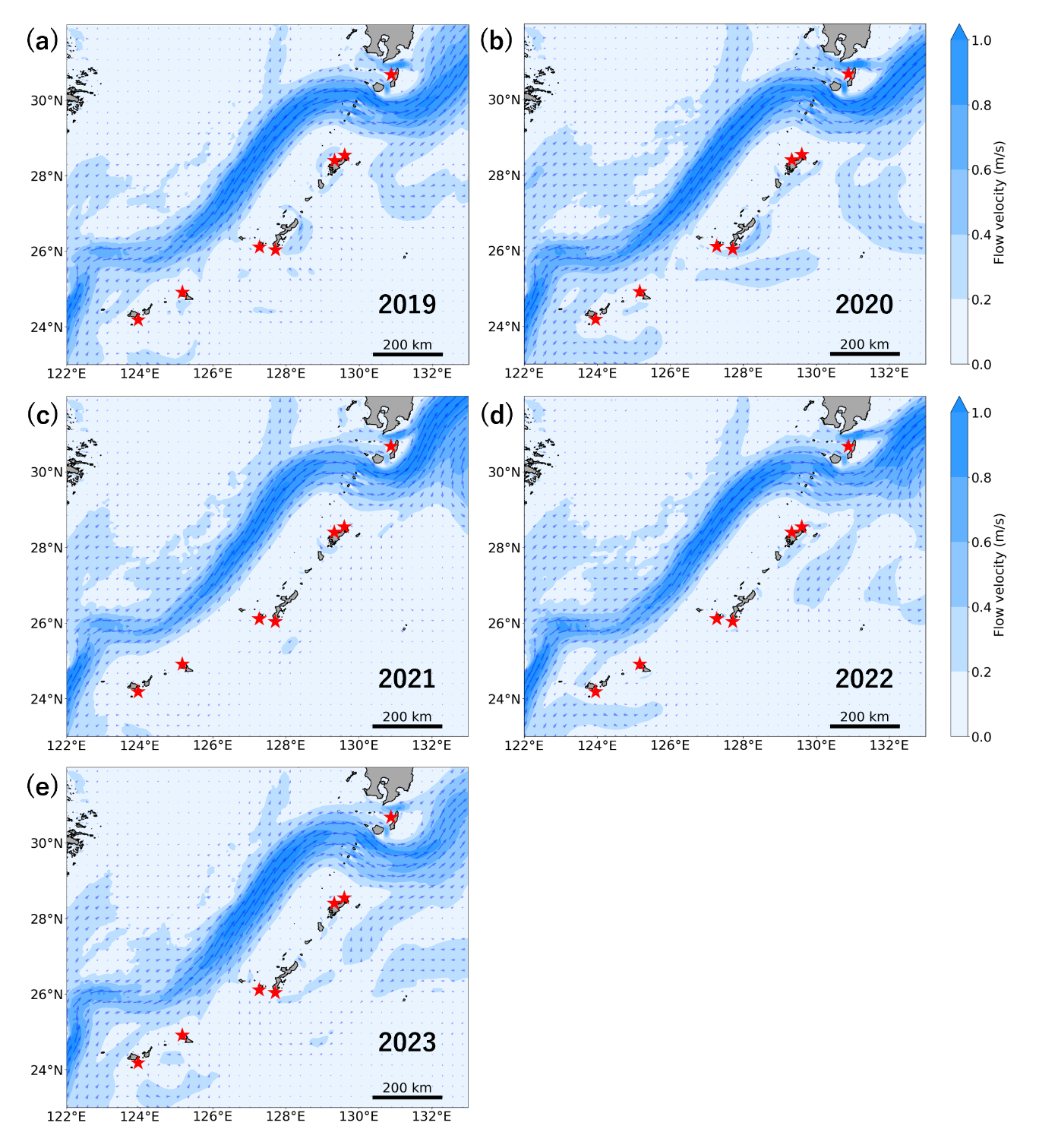


Fig. S5. Annual mean current flows at 0 m depth during the coral spawning period (May to August), derived from the ocean model JCOPE-T. Colours represent velocities, and arrows indicate current directions. The year is shown in the bottom-right corner of each panel.

**References**

Miyazawa, Y. et al. Assimilation of high-resolution sea surface temperature data into an operational nowcast/forecast system around Japan using a multi-scale three-dimensional variational scheme. *Ocean Dyn.* **67**, 713–728 (2017).

Miyazawa, Y. et al. Temperature profiling measurements by sea turtles improve ocean state estimation in the Kuroshio-Oyashio Confluence region. *Ocean Dyn.* **69**, 267–282 (2018).
